# Postoperative oscillatory brain activity as an add-on prognostic marker in diffuse glioma

**DOI:** 10.1007/s11060-019-03386-7

**Published:** 2020-01-17

**Authors:** Vera Belgers, Tianne Numan, Shanna D. Kulik, Arjan Hillebrand, Philip C. de Witt Hamer, Jeroen J. G. Geurts, Jaap C. Reijneveld, Pieter Wesseling, Martin Klein, Jolanda Derks, Linda Douw

**Affiliations:** 1 Anatomy & Neurosciences, Amsterdam Neuroscience, Amsterdam UMC, Vrije Universiteit Amsterdam, De Boelelaan 1117, 1081 HV Amsterdam, Netherlands; 2 Brain Tumor Center, Cancer Center Amsterdam, Amsterdam UMC, Vrije Universiteit Amsterdam, De Boelelaan 1117, 1081 HV Amsterdam, Netherlands; 3 Clinical Neurophysiology and MEG Center, Amsterdam Neuroscience, Amsterdam UMC, Vrije Universiteit Amsterdam, De Boelelaan 1117, 1081 HV Amsterdam, Netherlands; 4 Neurosurgery, Amsterdam Neuroscience, Amsterdam UMC, Vrije Universiteit Amsterdam, De Boelelaan 1117, 1081 HV Amsterdam, Netherlands; 5 Neurology, Amsterdam Neuroscience, Amsterdam UMC, Vrije Universiteit Amsterdam, De Boelelaan 1117, 1081 HV Amsterdam, Netherlands; 6 Pathology, Amsterdam Neuroscience, Amsterdam UMC, Vrije Universiteit Amsterdam, De Boelelaan 1117, 1081 HV Amsterdam, Netherlands; 7 Medical Psychology, Amsterdam Neuroscience, Amsterdam UMC, Vrije Universiteit Amsterdam, De Boelelaan 1117, 1081 HV Amsterdam, Netherlands; 8 Department of Radiology, Athinoula A. Martinos Center for Biomedical Imaging, Massachusetts General Hospital, 149 13th street, Charlestown, MA USA

**Keywords:** Glioma, Progression-free survival, Overall survival, Magnetoencephalography (MEG), Beamforming

## Abstract

**Introduction:**

Progression-free survival (PFS) in glioma patients varies widely, even when stratifying for known predictors (i.e. age, molecular tumor subtype, presence of epilepsy, tumor grade and Karnofsky performance status). Neuronal activity has been shown to accelerate tumor growth in an animal model, suggesting that brain activity may be valuable as a PFS predictor. We investigated whether postoperative oscillatory brain activity, assessed by resting-state magnetoencephalography is of additional value when predicting PFS in glioma patients.

**Methods:**

We included 27 patients with grade II–IV gliomas. Each patient’s oscillatory brain activity was estimated by calculating broadband power (0.5–48 Hz) in 56 epochs of 3.27 s and averaged over 78 cortical regions of the Automated Anatomical Labeling atlas. Cox proportional hazard analysis was performed to test the predictive value of broadband power towards PFS, adjusting for known predictors by backward elimination.

**Results:**

Higher broadband power predicted shorter PFS after adjusting for known prognostic factors (n = 27; HR 2.56 (95% confidence interval (CI) 1.15–5.70); *p* = 0.022). Post-hoc univariate analysis showed that higher broadband power also predicted shorter overall survival (OS; n = 38; HR 1.88 (95% CI 1.00–3.54); *p* = 0.038).

**Conclusions:**

Our findings suggest that postoperative broadband power is of additional value in predicting PFS beyond already known predictors.

**Electronic supplementary material:**

The online version of this article (10.1007/s11060-019-03386-7) contains supplementary material, which is available to authorized users.

## Introduction

Glioma prognosis is currently determined by factors such as molecular tumor subtype, tumor grade, performance status and age [1]. Still, there is a large variability in progression-free and overall survival (PFS and OS, respectively) that cannot be explained by these prognostic factors [2, 3]. An accurate prognosis is essential for medical decision-making [4]. In order to develop better prognostics, we need to expand our current knowledge of factors that contribute to tumor growth and progression.

One proposed factor associated with glioma growth is neuronal activity. By introducing light-sensitive proteins into the brain, neurons can be activated through light (optogenetics) [5]. This technique was applied in a murine model, showing that the stimulation of neurons promotes glioma growth [6]. This association between neuronal activity and glioma growth is also present without (possibly non-physiological) stimulation through optogenetics: in vitro, spontaneous aberrant neuronal activity is observed even in the earliest stages of glioma [7]. The protein neuroligin-3 (NLGN3) has been suggested to play a key role in glioma growth [8]. NLGN3 is a synaptic adhesion molecule and is important for normal synaptic function and brain plasticity [8]. In the murine model, increased neuronal activity promotes the cleavage and secretion of NLGN3, which then triggers mitosis—and thereby tumor growth—in gliomas [8].

**Fig. 1:**
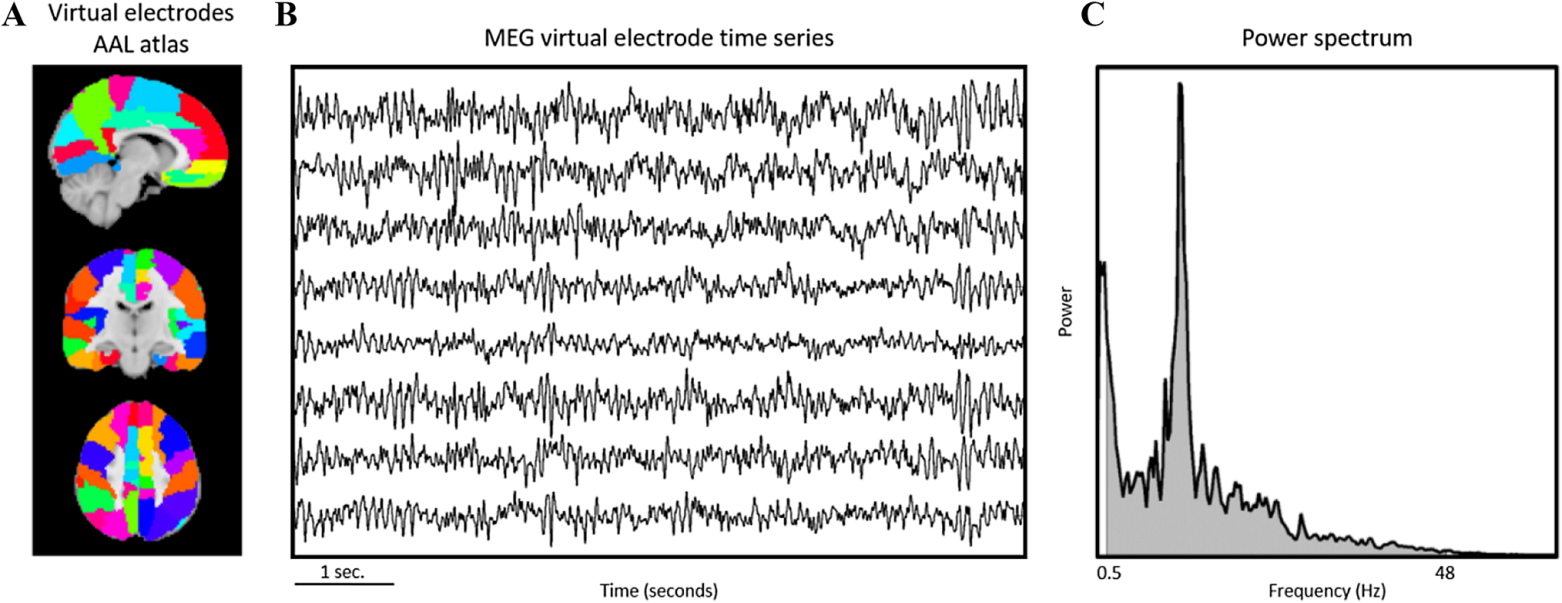
Broadband power calculated from magnetoencephalography time series: **a** Time series were estimated, using beamforming, for virtual electrodes placed at the centroids of regions in the automated anatomical labeling (AAL) atlas. **b** Exemplar broadband time series. **c** The power spectrum for each time series was obtained using a fast Fourier transform, and then averaged. Broadband power was calculated as the area under the curve between 0.5 and 48 Hz (grey surface)

Neuronal activity in patients may be assessed non-invasively using magnetoencephalography (MEG) or electroencephalography (EEG). MEG records the magnetic fields induced by synchronous postsynaptic neuronal activity and, unlike EEG, is not perturbed by the skull and scalp [9, 10]. The MEG recordings can be projected to source-space, resulting in estimated time series of localized neuronal activity [11]. ‘Power’ is the squared amplitude of the time series and can be analyzed within particular frequency bands or across all frequencies (broadband power; see Fig. 1). Broadband power best correlates with neuronal spiking patterns in patients undergoing both non-invasive neurophysiological measurements as well as single-cell recordings using intracranially placed electrodes [12]. We recently reported on the association between broadband power and PFS in twenty-four newly-diagnosed glioma patients: lower preoperative broadband power predicted longer PFS [13]. While this suggests that broadband power may be a useful prognostic tool in glioma, prognosis based on information after resection might be more reliable than preoperatively measured broadband power. Also, postoperative broadband power can be combined with information on molecular subtype and histology, which are important prognosis predictors. Additionally, postoperative broadband power might logistically be easier to obtain.

To gain a deeper understanding of predictors of PFS in the postoperative clinical setting, we investigated whether higher postoperative broadband power predicts shorter PFS in a cohort of glioma patients. For clinical relevance, we adjusted for already known predictors thereby investigating the added value of broadband power as a predictor. As a post-hoc analysis, we investigated the relationship between broadband power and OS.

## Methods

### Patients

This retrospective study cohort, which combined several datasets of patients previously reported on, consisted of de novo glioma patients who underwent tumor resection at the Brain Tumor Center Amsterdam [part of Amsterdam University Medical Center, location Vrije Universiteit (VUmc)] between 2010 and 2017 [13, 14]. Inclusion criteria for these studies were (1) age over 17 years, (2) histopathologically confirmed glioma grade II or higher (as defined by the 2016 classification of the World Health Organization [1]), (3) tumor resection as part of their treatment, and (4) no history of neurologic or psychiatric disease. For the current investigation, only patients were included who had undergone a resting-state MEG after tumor resection. Molecular markers (isocitrate dehydrogenase (IDH) mutation and 1p19q codeletion) were determined in the context of routine clinical care, and therefore not available for all patients [1].

A cohort of healthy controls, previously described in the setting of studies on multiple sclerosis, was used to normalize indices of broadband power [15, 16]. The healthy subjects were group-level matched to the glioma patients with respect to age, as we know this can affect brain activity [17, 18]. Data collection in both patients and healthy controls was approved by the ethical review board of the VUmc. Informed consent was obtained before participation.

In the glioma patients, PFS was defined as the time (in weeks) between the MEG recording and the date of radiological or clinical progression, as determined by a multidisciplinary tumor board including a neuroradiologist, neurosurgeon, radiation oncologist, medical oncologist and a neurologist. Patients without progression in the follow-up period (until December 2018) were censored after the last contact.

### Magnetoencephalography

MEG data were obtained using a 306-channel whole-head system (Elektra Neuromag Oy, Helsinki, Finland) with a sampling frequency of 1250 Hz. Brain activity was recorded for 5 min in a magnetically shielded room (VacuumSchmelze GmbH, Hanua, Germany) during an eyes-closed resting-state in supine position. Patients were instructed to lay still, keep their eyes closed and to stay awake. From the raw data, malfunctioning channels were excluded after visual inspection and artefacts were filtered offline using the temporal extension of Signal Space Separation in MaxFilter software (Elektra Neuromag Oy, version 2.2.15) [19, 20]. A 3D digitizer (Fastrak, Polhelmus, Colchester, VT, USA) was used to digitize four of five head localization coils and scalp shape. These data were co-registered to the patient’s anatomical MRI using a surface-matching approach, to enable projection to the anatomical space. In the 78 cortical regions of the Automated Anatomical Labeling (AAL) atlas, time series were reconstructed through beamforming (see [21–23] for details) and then divided into consecutive epochs (time segments) of 3.27 s [23]. For each patient, 56 artifact-free epochs were included for analysis, 52 epochs for the healthy controls [19]. No detrending or downsampling was performed, all analyses were performed on data with a sample frequency of 1250 Hz. A fast Fourier transform was performed to obtain the power spectrum for each epoch, and broadband power (0.5–48 Hz) was calculated. Next, broadband power was averaged over all epochs and all regions per subject. These calculations were performed using Matlab (Mathworks, Natick, MA, USA, version R2012a). The broadband power values were converted to z-scores based on the mean and standard deviation of the healthy subjects.

### Statistical analyses

Data were analyzed using IBM SPSS Statistics for Windows (IBM Corp., Armonk, NY, USA, version 22.0.0.0). P-values smaller than 0.05 were considered statistically significant. Group differences between healthy controls and patients were assessed with Student’s t tests for continuous variables and χ^2^-test for categorical data.

To test our hypothesis on the additional prognostic value of postoperative broadband power towards PFS, we created a Cox proportional hazards model. We adjusted this analysis for age (continuous), Karnofsky performance score (KPS; ordinal), presence of epilepsy (dichotomous), WHO tumor grade (grade II or III/IV) and IDH-mutation/1p19q-status (IDH-mutated (IDH-mut) and 1p19q-codeleted, IDH-mut but non-codeleted, or IDH-wildtype (IDH-wt)) [1, 24–29]. Predictors in the Cox proportional hazards model were selected by backward elimination (cutoff *p* < 0.1). To avoid overfitting, the maximum number of covariates was determined by dividing the number of patients with progression by six [30]. If more covariates would have been selected through backward elimination, then the least significant predictors were to be eliminated. Robustness of the model was assessed by performing leave-one-out analyses, each time repeating the analysis excluding one patient. Kaplan–Meier plots were rendered for visualization.

Finally, we examined whether our current results were driven by the patients who were previously studied in the presurgical phase of their disease [13]. We did this by dividing the cohort into two subgroups (previously/not previously included by Derks and colleagues) [13]. Cox univariate analyses were then performed in these two groups separately.

## Results

### Participant characteristics

**Fig. 2:**
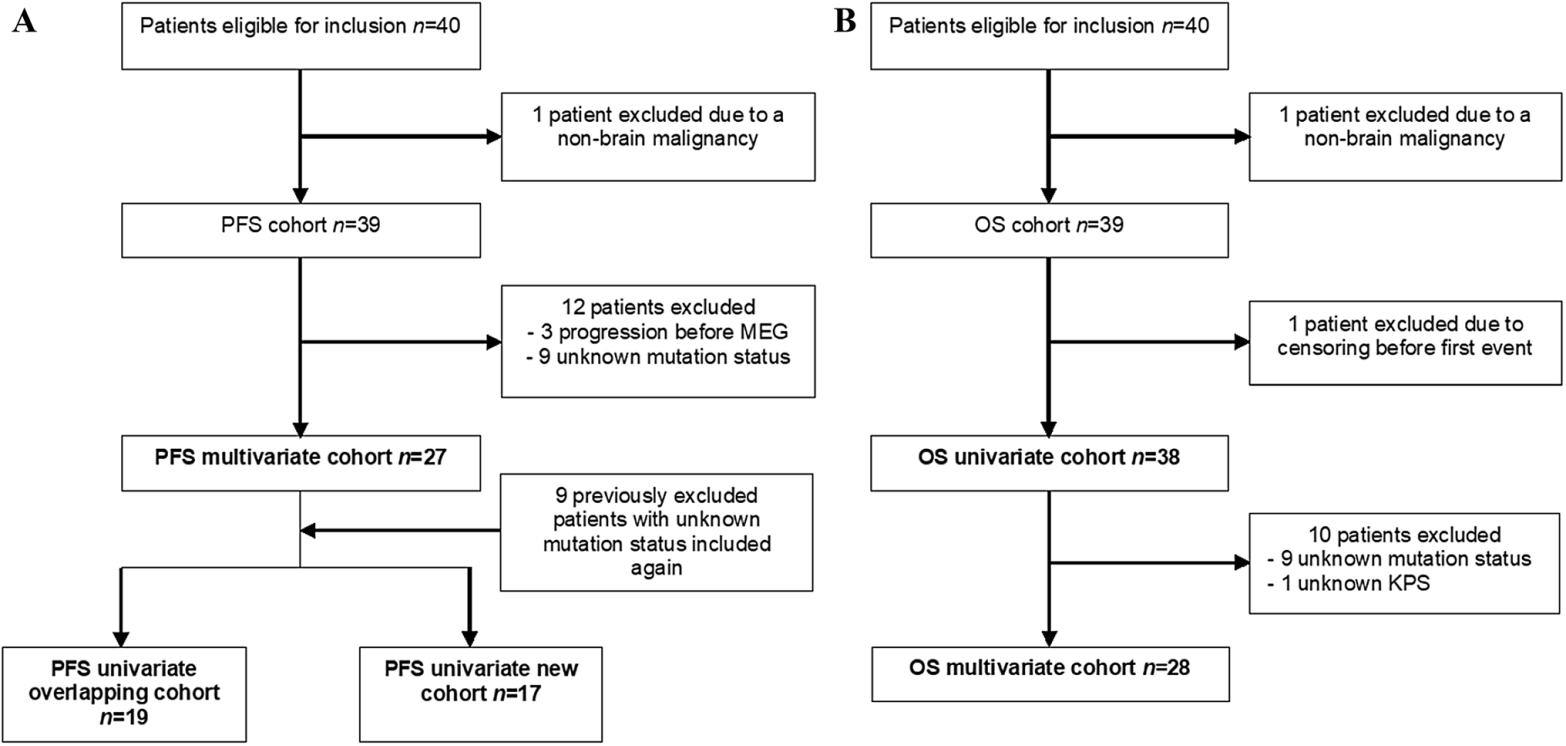
Flow chart of inclusion for the **a** PFS cohort (subsequently divided based on whether patients were previously analyzed by Derks et al. [13]) and **b** OS cohort

Forty patients met the initial inclusion criteria. One patient was excluded due to a comorbid non-brain malignancy, receiving chemotherapy during MEG recording and follow-up. Nine patients had to be excluded from the main analysis due to unknown IDH/1p19q status (these patients were included in the exploratory analysis of OS). Three additional patients were excluded due to progression before MEG (Fig. 2). The final cohort consisted of 27 patients (see Fig. 2 and Supplementary Table 1) and 27 matched healthy controls.

**Table 1:** Baseline characteristics study cohort

	Patients (n = 27)	Healthy controls (n = 27)
Mean age (SD)	41.6 (12.8)	46.7 (4.7)
# Male (%)	18 (66.7)	10 (37.0)
Mean z-score broadband power (SD)	0.0 (0.9)	0.0 (1.0)
# Patients with grade II glioma (%)	13 (48.1)	
# Patients with grade III glioma (%)	7 (25.9)	
# Patients with grade IV glioma (%)	7 (25.9)	
*IDH/1p19q status*		
# Tumors with IDH-mut/1p19q codeletion (%)	6 (22.2)	
# Tumors with IDH-mut/1p19q non-codeletion (%)	14 (51.9)	
# Tumors with IDH-wt (%)	7 (25.9)	
# Patients with epilepsy (%)	21 (77.8)	
# Patients using AEDs (%)	21 (77.8)	
# Patients treated with radiotherapy (%)	24 (88.9)	
# Patients treated with chemotherapy (%)	17 (63.0)	
Median KPS [range]	100 [70–100]	
Median # weeks between resection and MEG [range]	22 [3–94]	
Median # weeks PFS or loss to follow-up [range]	75 [0–376]	
Median # weeks of OS or loss to follow-up (n = 38, [range])	276 [6–372]	
# Patients with tumor progression (%)	20 (74.1)	
# Patients who died (%)	11 (40.7)	

*AED* antiepileptic drug, *IDH* isocitrate dehydrogenase, *KPS* Karnofsky performance score, *OS* overall survival, *PFS* progression-free survival, *SD* standard deviation, *WT* wildtype

In total, 20 patients showed progression during follow-up, with a median PFS of 35 (range 0–249) weeks (see Table 1 for detailed participant characteristics). There were no differences between patients and healthy controls in terms of broadband power (*t*(55) = 0.250, *p* = 0.803) or age (*t*(52) = − 1.94, *p* = 0.058). Median time between tumor resection and postoperative MEG recording was 19 weeks (range 3–94). Broadband power and time since tumor resection were not correlated (Kendall’s τ = 0.082, *p* = 0.467).

### Higher broadband power relates to shorter progression-free survival

**Fig. 3:**
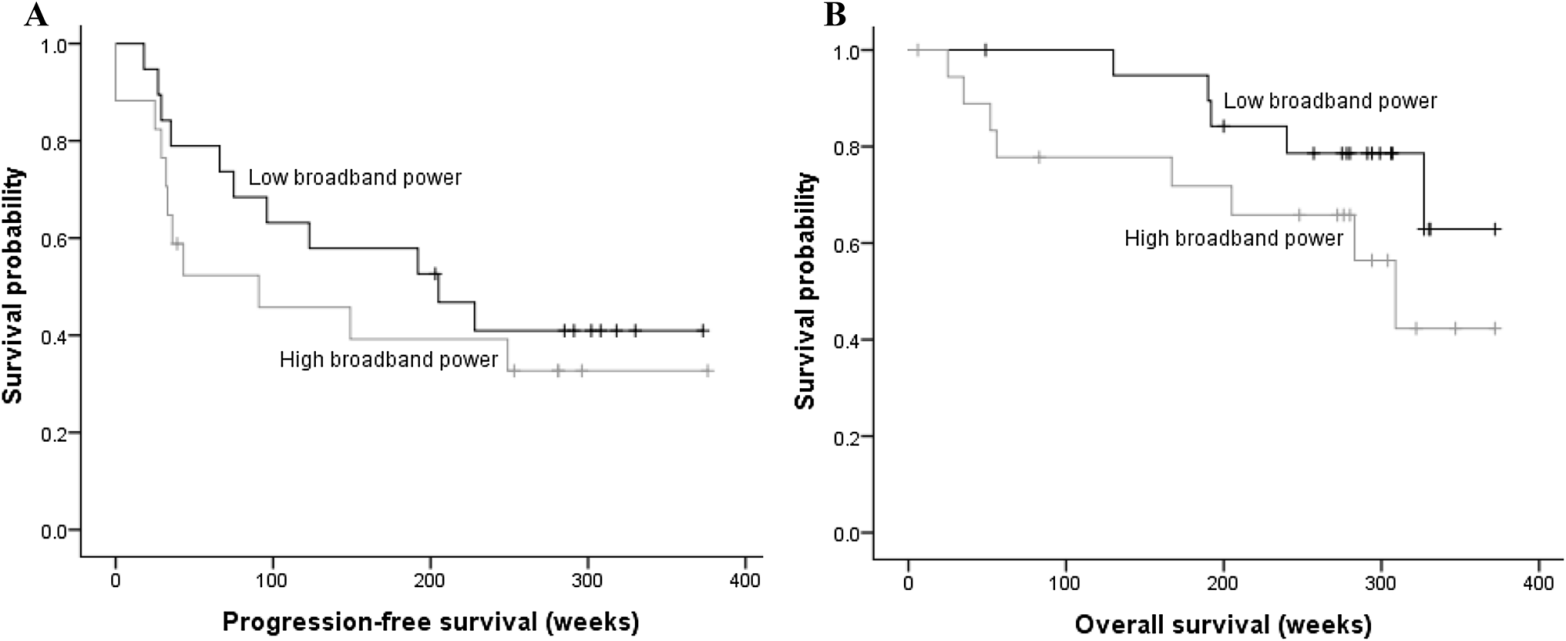
Kaplan–Meier curves for the **a** PFS cohort (n = 27) and **b** OS cohort (n = 38) as a function of broadband power, based on a median split in the patient population

Backward elimination resulted in a model with broadband power, age and IDH/1p19q status as significant predictors. This indicates that broadband power, age and IDH/1p19q status were significantly predictive of PFS, while tumor grade, epilepsy and KPS were not. In this model, increased broadband power predicted significantly shorter PFS with a hazard ratio (HR) of 2.56 (*p* = 0.022; Fig. 3; Table 2). IDH-wt tumors had shorter PFS than IDH-mut tumors (HR 52.44; *p* = 0.001), as expected. Contrary to our expectations, older age predicted longer PFS (HR 0.93; *p* = 0.032). Post-hoc analyses showed that age alone did not significantly predict PFS (HR 0.99; 95% CI 0.96–1.03; *p* = 0.737), moreover, no correlation between age and PFS was found (r_s_ = 0.14, *p* = 0.66).

**Table 2:** Cox proportional hazards analyses

Model	Dependent variable	Predictor	HR (95% CI)	p-value
Multivariate	PFS	Broadband power	2.56 (1.15–5.70)	0.022*
		Age	0.93 (0.91–0.97)	0.032*
		IDH-mut/codel	Reference	–
		IDH-mut/non-codel	13.18 (1.61–17.02)	0.016*
		IDH-wt	52.44 (5.28–521.19)	0.001*
Post-hoc univariate	OS	Broadband power	1.94 (1.04–3.62)	0.038*
Post-hoc multivariate	OS	Broadband power	3.74 (1.23–11.36)	0.020*
		Age	1.05 (0.97–1.23)	0.30
		IDH-mut/codel	Reference	–
		IDH-mut/non-codel	5641.31 (0–1.16 × 1042)^a^	0.85
		IDH-wt	5.19 × 1012 (0–4.19 × 1010)^a^	0.80
		KPS	1.06 (0.97–1.15)	0.20
		Epilepsy	125.60 (2.20–7159.94)	0.019*
		Grade II	Reference	–
		Grade III/IV	0.010 (0–1.42 × 10^36^)	0.92

*95% CI* 95% confidence interval, *HR* hazard ratio, *IDH* isocitrate dehydrogenase, *IDH-mut/codel* IDH-mutant, *1p19q* codeleted, IDH-mut/non-codel IDH-mutant, 1p19q non-codeleted, *IDH-wt* IDH-wildtype, *KPS* Karnofsky performance score

*p < 0.05

^a^No people died in the reference group

### Robustness of results

In the leave-one-out validation, broadband power was predominantly significant as a predictor of PFS, while revealing statistically insignificant results four times (*p* = 0.054; 0.061; 0.10; 0.11; 14.8%), as was age (*p* = 0.051; 0.072; 0.074; 0.14), indicating some model instability. IDH/1p19q status remained a statistically significant predictor in all models.

### Consistency across cohorts

We split the cohort based on whether patients had previously been analyzed or not (see Fig. 2) [13]. We did not adjust for any other predictors as this was an exploratory univariate analysis. We could therefore include patients with an unknown mutation status, resulting in a cohort of 36 patients (see Fig. 2). This resulted in two groups of roughly equal size: 19 patients overlapped with the cohort of 24 patients analyzed in the presurgical phase by Derks et al., and the new cohort consisted of 17 patients. Broadband power did not significantly predict PFS in either the known (HR 1.61; *p* = 0.288; Table 3) or new (HR 1.95; *p* = 0.117) cohort, see Table 3. However, comparable hazard ratios for higher broadband power relating to shorter PFS were observed in both groups (with an even higher HR in the completely new cohort), suggesting that our results were not driven by the previously analyzed patients only, but instead were comparable in a new sample of patients.

**Table 3:** Cox proportional hazards analyses after dividing the cohort

Model	Dependent variable	Predictor	Total # of patients	HR (95% CI)	p-value
Univariate, known cohort	PFS	Broadband power	19	1.61 (0.67–3.84)	0.288
Univariate, new cohort	PFS	Broadband power	17	1.95 (0.85–4.48)	0.117

*95% CI 95%* confidence interval*, HR* hazard ratio, *PFS* progression-free survival

### Post-hoc analysis: higher broadband power relates to shorter OS

Even though overall survival is less intuitively related to tumor growth speed than PFS (as second line treatment effects may compound confounding factors later in the disease), we explored the predictive value of broadband power towards OS in post-hoc analyses. In this cohort of 38 patients, 13 patients died within follow-up, with a median OS of 167 (range 25–327) weeks. In the PFS analyses, the maximal number of covariates was set to one per six cases of progression, or in this case, death [30]. As only thirteen patients had died, the OS multivariate model did not meet this criterium, resulting in an overfitted model. We therefore decided to analyze both a multivariate model to adjust for known predictors, as well as a univariate model to explore the direct relationship between broadband power and OS.

The univariate cohort consisted of 38 patients (see Fig. 2). This model showed broadband power to be predictive of OS (HR 1.94; *p* = 0.038; see Table 2). The multivariate cohort was smaller as we excluded 10 patients due to unknown data, resulting in a cohort of 28 patients (see Fig. 2). We selected the most important predictors by performing backward elimination, which meant that in this case, all predictors were included. This model showed broadband power (HR 3.74; *p* = 0.020) and epilepsy (HR 125.6; *p* = 0.019) to be significant predictors, see Table 2. It should be noted, however, that no patients with IDH-mutant/1p19q codeleted glioma died within our follow-up time frame, and that the epilepsy subgroup was very small. Both factors possibly attributed to the exorbitant hazard ratios.

We assessed the robustness of both models by performing leave-one-out analyses, repeating all analyses, each time excluding one patient. This resulted in a univariate non-significant model six times (*p* = 0.051; 0.061; 0.064; 0.076; 0.093; 0.203; 15.8%). The multivariate model revealed statistically non-significant results three times (*p* = 0.059; 0.064; 0.128; 10.7%).

### Exploratory analysis: sensor-level brain activity is not predictive of PFS

MEG recordings were projected to source-level, resulting in estimated time series of localized neuronal activity [11]. However, sensor-level measurements also provide an estimate of the underlying brain activity. Source-level analysis has a superior spatial resolution and improves the signal-to-noise ratio when techniques such as beamforming are used, thereby improving the estimates of brain activity (see e.g. [31, 32]). Reconstructing source-level data, however, is a time-consuming task. We therefore explored whether sensor-level information from gradiometers could also predict PFS. Sensor-level broadband power did not predict PFS in the univariate (HR 1.240; p = 0.535) or multivariate (HR 1.003; p = 0.993) analysis (see Supplementary Table 2).

## Discussion

Our results demonstrate that brain activity in glioma patients as measured by MEG is associated with PFS when adjusting for other known predictors (age, tumor grade, KPS, epilepsy and IDH/1p19q status). According to our results, assessment of broadband power could thus improve the current prognosis prediction. Although we did not directly measure tumor growth, these findings also support the hypothesis that higher levels of neuronal activity are associated with faster tumor growth. Activity recorded at the sensor-level did not predict PFS, emphasizing the importance of reconstructing activity at the source-level [31, 32].

Older age was associated with a slightly longer PFS (HR 0.93), whereas we expected age to predict a slightly shorter PFS (previously reported HR 1.02–1.06) [28]. This could be due to our small sample size, or to a possible selection bias that resulted in above average vitality in our older patients.

Results from the post-hoc analyses also suggest a relationship between broadband power and overall survival. The univariate model showed broadband power to be predictive of OS, the multivariate model verified this connection. Even though this multivariate model was overfitted and thus had limited statistical power, it suggests that the predictive value of broadband power cannot be explained by other predictors and is an independent predictor of OS.

In our patients, broadband power was assessed after tumor resection, which is a relevant phase of the disease in terms of treatment strategy. Part of the current cohort has been reported on with respect to preoperative broadband power, showing that higher oscillatory activity before tumor resection significantly predicts PFS [13]. Hazard ratios for broadband power were similar in the separate analyses of the previously analyzed cohort and the new cohort, suggesting that our findings are robust, both in terms of time point (before or after tumor resection) and cohorts. Although broadband power did not reach significance in predicting PFS in the two small subgroups (likely due to reduced statistical power), hazard ratios were comparable to those found for the entire cohort. This indicates that the current results were not driven by those patients already included in our previous study but may be seen as a replication and extension of those results.

Broadband power was based on all AAL regions, because Derks et al. showed similar results in the preoperative setting for tumor regions specifically and when including all AAL regions [13]. From a brain network perspective, glioma and the resection of glioma affects the entire brain network and it is therefore reasonable to expect global alterations in broadband power [33, 34].

### Clinical implications

Glioma prognosis and treatment are determined preoperatively and confirmed or adapted postoperatively, based predominantly on histopathology, molecular tumor subtyping, radiological imaging and patient condition. Our results suggest that broadband power could contribute to assessment of prognosis in the postoperative disease phase.

Additionally, broadband power may speculatively be useful in dynamically monitoring disease course. If broadband power is indeed a valid proxy of the neuronal activity that leads to accelerated tumor growth, (forthcoming) tumor progression may conceivably be detected with broadband power before radiological or clinical progression is observable [35]. If so, this would be particularly relevant in the context of pseudoprogression. Pseudoprogression occurs in approximately 10–30% of patients treated for gliomas, and is difficult to distinguish from real tumor progression [36]. With conventional MRI, an estimated 37% of diagnosed progressive disease cases are actually (at least partly) pseudoprogression [36]. Consequently, current guidelines discourage diagnosing tumor progression within the first 3 months posttreatment [37]. Due to the aggressive nature of (in particular) glioblastomas, however, progression might very well be seen within these first months, underlining the need for better, non-invasive markers of (early) progression. Larger prospective studies are necessary to investigate the value of broadband power in this context.

A foreseen barrier towards clinical implementation of our current results is the fact that MEG is costly and not widely available in hospitals around the world, although new technical developments may change this situation rapidly [38]. For now, a more feasible alternative could be to use EEG, as it is widely available and less costly. Although EEG is less accurate and more prone to artifacts than MEG, it might be worth investigating whether EEG also reliably measures broadband neuronal power and predicts PFS.

Even more valuable than improving survival prediction and monitoring, is the possibility of developing new treatment targets based on the mechanism hypothesized to underlie the predictive value of broadband power for PFS. NLGN3 has been suggested to play a key role in glioma growth and might therefore be a viable treatment target [8]. Indeed, gliomas fail to grow in NLGN3 knockout mice, while blocking NLGN3 release prevents tumor growth in animals [6]. However, these inhibitors are currently not suitable for human use. Therefore, a more feasible treatment target might be neuronal activity, which might be reduced through antiepileptic drugs (AEDs) as these could diminish neuronal activity, or through non-invasive inhibitory stimulation using transcranial magnetic stimulation (TMS) or transcranial direct/alternating current stimulation (tDCS/tACS) [35–42]. Clinical trials are necessary to further explore the therapeutic benefits of these targets.

### Limitations

First, although very well-characterized, the study cohort should be considered small, so caution must be applied when interpreting our results. The robustness of the results was evaluated with leave-one-out analyses, showing a 11–16% model instability, probably due to the low sample size. Nevertheless, our study results are in line with previous findings [13].

Furthermore, the study population does not accurately reflect the general diffuse glioma patient population: our patients generally had prognostically favorable tumors, were relatively young and had high performance status. This selection bias possibly resulted in the surprising finding that older age was associated with longer PFS. Although the effect was small (HR 0.95), it was significant, underlining that we should interpret our findings with caution when extrapolating to the general diffuse glioma population.

Most patients used an AED. Even though AED dosing was titrated in order to achieve seizure freedom and may thus not reach levels necessary for the dose-dependent lowering of neuronal activity, we cannot exclude the possibility that AED use influenced our results [43].

Last, we used broadband activity as a proxy of neuronal firing. Although we did not directly measure neuronal activity, assessment through MEG remains its most accurate non-invasive proxy [10, 12].

## Conclusions

Postoperative broadband power as measured with MEG emerges as a valuable addition to currently known predictors of PFS and OS in glioma patients. Larger prospective cohort studies to investigate the relationship between broadband power and tumor growth are needed to translate these findings to clinical practice. Our findings may be a next step towards improving accuracy of glioma prognosis and disease monitoring, and may offer new leads towards new glioma therapy.

## Electronic supplementary material

Below is the link to the electronic supplementary material.

Supplementary file1 (DOCX 35 kb)
