# Supplementary material for: Postoperative oscillatory brain activity as an add-on prognostic marker in diffuse glioma
Source: J Neurooncol. 2020 Jan 17;147(1):49–58. doi: 10.1007/s11060-019-03386-7 (PMC7075827; doi:10.1007/s11060-019-03386-7)
Supplement: Supplementary file 1 — Supplementary file1 (DOCX 35 kb) [file 11060_2019_3386_MOESM1_ESM.docx]

**Supplementary Materials**

**Postoperative oscillatory brain activity as an add-on prognostic marker in diffuse glioma**

Vera Belgers^1,2^, Tianne Numan^1,2^, Shanna D. Kulik^1,2^, Arjan Hillebrand^3^, Philip C. de Witt Hamer^2,4^, Jeroen J.G. Geurts^1^, Jaap C. Reijneveld^2,5^, Pieter Wesseling^2,6^, Martin Klein^2,7^, Jolanda Derks^1,2^, Linda Douw^1,2,8^

^1^Amsterdam UMC, Vrije Universiteit Amsterdam, Anatomy & Neurosciences, Amsterdam Neuroscience, De Boelelaan 1117, Amsterdam, Netherlands

^2^Amsterdam UMC, Vrije Universiteit Amsterdam, Brain Tumor Center, Cancer Center Amsterdam, De Boelelaan 1117, Amsterdam, Netherlands

^3^Amsterdam UMC, Vrije Universiteit Amsterdam, Clinical Neurophysiology and MEG Center, Amsterdam Neuroscience, De Boelelaan 1117, Amsterdam, Netherlands

^4^Amsterdam UMC, Vrije Universiteit Amsterdam, Neurosurgery, Amsterdam Neuroscience, De Boelelaan 1117, Amsterdam, Netherlands

^5^Amsterdam UMC, Vrije Universiteit Amsterdam, Neurology, Amsterdam Neuroscience, De Boelelaan 1117, Amsterdam, Netherlands

^6^Amsterdam UMC, Vrije Universiteit Amsterdam, Pathology, Amsterdam Neuroscience, De Boelelaan 1117, Amsterdam, Netherlands

^7^Amsterdam UMC, Vrije Universiteit Amsterdam, Medical Psychology, Amsterdam Neuroscience, De Boelelaan 1117, Amsterdam, Netherlands

^8^Department of Radiology, Athinoula A. Martinos Center for Biomedical Imaging, Massachusetts General Hospital, 149 13^th^ street, Charlestown, MA, USA

**Corresponding author** L. Douw; [l.douw@amsterdamumc.nl](mailto:l.douw@amsterdamumc.nl)

**Journal of Neuro-Oncology**

**Supplementary Materials**

MEG Data Acquisition

MEG data were obtained using a 306-channel whole-head MEG system (Elektra Neuromag Oy, Helsinki, Finland) with a sampling frequency of 1250 Hz. Two filters were applied online: a 410 Hz anti-aliasing filter and a high pass filter of 0.1 Hz. Brain activity was recorded for 5 minutes in a magnetically shielded room (VacuumSchmelze GmbH, Hanua, Germany) during an eyes-closed resting state in supine position. Patients were instructed to lay still, keep their eyes-closed and to stay awake. This was monitored by the examiner.

Malfunctioning channels (number ranging between 1-12 (median 11)) were visually identified in the data following cross-validation Signal Space Separation (xSSS)[1]. Artefacts were subsequently removed offline using the temporally extended Signal Space Separation (tSSS) in MaxFilter software (Elektra Neuromag Oy, version 2.2.15)[2–4], using a sliding window of 10 seconds and sub-space correlation limit of 0.9. These spatially filtered data were then temporally filtered using a single-pass FIR filter in MaxFilter, using a Kaiser window with an order of 5 times the sample frequency, divided by the corner-value of the low (48 Hz) or high pass (0.5 Hz) filter, and with an attenuation of 60 dB at 1.5 times the low-pass corner or 0.7 times the high-pass corner, respectively.

A 3D digitizer (Fastrak, Polhelmus, Colchester, VT, USA) was used to digitize four of five head localization coils and scalp shape (~500 points). To enable projection to the anatomical space, these data were co-registered to the patient’s anatomical MRI using a surface-matching approach with an accuracy of approximately 4 mm.

MEG Preprocessing

A single sphere model was fitted to the scalp surface that was extracted from the patient’s MRI. Segmentation was performed using FieldTrip (version 31-05-2018; function *ft_volumesegment*) resulting in a binary segmentation of the scalp.

Time series of neuronal activity were reconstructed through beamforming for the 78 cortical regions of the Automated Anatomical Labeling (AAL) atlas (see [5, 6] for details). The data covariance matrix, based on the broadband filtered time series (mean duration = 285 seconds; range 188-327 seconds), was used with a unity noise covariance matrix and the lead fields for an equivalent current dipole to compute beamformer weights for different locations [7, 8]. The data covariance matrix was regularized using singular value truncation, using the default setting of 1.0e-06. Optimum orientation of the equivalent current dipole was found using singular value decomposition [8]. The broadband data were subsequently, and sequentially, projected through the beamformer weights for the centroid within each AAL region, resulting in source-level broadband time series for all regions in the AAL atlas.

The calculated time series were divided into consecutive epochs (time segments) of 3.27 seconds. For each patient, 56 artifact-free epochs were included for analysis, for each healthy control, 52 epochs were included, based on the lowest number of available artifact-free epochs in each group respectively. A fast Fourier transform was performed to obtain the power spectrum for each epoch using the *fft* function in Matlab with a window size of 4096, and broadband power (0.5-48 Hz) was calculated. Next, broadband power was averaged over all epochs and all regions per subject. These calculations were performed using Matlab (Mathworks, Natick, MA, USA, version R2012a). The broadband power values were converted to z-scores based on the mean and standard deviation of the healthy subjects.

**Supplementary tables**

**Supplementary table 1** Detailed patient information

| Patient | Sex | Age | Mutation status | Grade | Epilepsy | AED | KPS |
| --- | --- | --- | --- | --- | --- | --- | --- |
| 1 | female | 43 | IDH WT | IV | no | - | 80 |
| 2 | male | 35 | IDH WT | IV | no | - | 90 |
| 3 | male | 66 | IDH WT | IV | no | - | 100 |
| 4 | male | 47 | IDH-mut/non-codel | III | no | - | 100 |
| 5 | female | 48 | IDH-mut/non-codel | II | yes | VPA | 100 |
| 6 | male | 29 | IDH-mut/non-codel | II | yes | LEV + PTH | 100 |
| 7 | female | 53 | IDH-mut/non-codel | II | yes | LEV | 100 |
| 8 | male | 30 | IDH-mut/non-codel | II | yes | LEV + PTH | 100 |
| 9 | male | 52 | IDH-mut/codel | II | yes | VPA + LAM | 100 |
| 10 | female | 28 | IDH-mut/non-codel | II | yes | LEV | 80 |
| 11 | male | 18 | IDH-mut/non-codel | II | yes | VPA + LEV | 100 |
| 12 | male | 43 | IDH-mut/non-codel | II | yes | VPA | 90 |
| 13 | male | 31 | IDH-mut/non-codel | II | yes | LEV + PTH | 100 |
| 14 | male | 36 | IDH-mut/codel | III | yes | LEV | 100 |
| 15 | female | 26 | IDH-mut/non-codel | III | yes | VPA | 80 |
| 16 | male | 27 | IDH-mut/non-codel | II | yes | VPA | 100 |
| 17 | male | 37 | IDH-mut/codel | III | yes | LEV | 90 |
| 18 | female | 37 | IDH-mut/non-codel | II | yes | LEV | 100 |
| 19 | male | 46 | IDH WT | IV | yes | VPA | 100 |
| 20 | male | 46 | IDH-mut/codel | III | yes | LEV | 100 |
| 21 | female | 67 | IDH-mut/non-codel | II | yes | VPA | 80 |
| 22 | female | 52 | IDH-mut/non-codel | II | yes | LEV | 80 |
| 23 | male | 28 | IDH-mut/codel | III | yes | LEV | 90 |
| 24 | male | 48 | IDH WT | IV | no | - | 100 |
| 25 | male | 63 | IDH WT | IV | no | - | 80 |
| 26 | male | 35 | IDH WT | IV | yes | LEV | 70 |
| 27 | female | 53 | IDH-mut/codel | III | yes | LEV | 80 |

AED Antiepileptic drug, IDH Isocitrate dehydrogenase, IDH-mut/codel IDH-mutant, 1p19q codeleted, IDH-mut/non-codel IDH-mutant, 1p19q non-codeleted, IDH-wt IDH-wildtype, KPS Karnofsky performance score, LAM Lamotrigine, LEV Levetiracetam, PTH Phenytoin, VPA Valproic acid

**Supplementary table 2** Cox proportional hazards analyses on sensor-level data

| Model | Dependent variable | Predictor | HR (95% CI) | P-value |
| --- | --- | --- | --- | --- |
| Post-hoc univariate | PFS | Sensor-level broadband power | 1.240 (0.629-2.446) | 0.535 |
| Post-hoc multivariate | PFS | Sensor-level broadband power | 1.003 (0.488-2.063) | 0.993 |
|  |  | Age | 0.961 (0.922-1.001) | 0.055 |
|  |  | IDH-mut/codel | Reference | - |
|  |  | IDH-mut/non-codel | 3.190 (0.254-40.107) | 0.369 |
|  |  | IDH-wt | 12.434 (0.810-190.793) | 0.070 |
|  |  | KPS | 0.968 (0.907-1.033) | 0.325 |
|  |  | Epilepsy | 0.124 (0.011-1.448) | 0.096 |
|  |  | Grade II | Reference | - |
|  |  | Grade III/IV | 0.303 (0.038-2.377) | 0.256 |

95% CI *95% confidence interval*, HR *hazard ratio,* IDH *isocitrate dehydrogenase,* IDH-mut/codel *IDH-mutant, 1p19q codeleted,* IDH-mut/non-codel *IDH-mutant, 1p19q non-codeleted,* IDH-*wt IDH-wildtype,* KPS *Karnofsky performance score*

**References**

[1] N. van Klink *et al.*, “Automatic detection and visualisation of MEG ripple oscillations in epilepsy,” *NeuroImage Clin.*, vol. 15, pp. 689–701, 2017.

[2] S. Taulu and J. Simola, “Spatiotemporal signal space separation method for rejecting nearby interference in MEG measurements Spatiotemporal signal space separation method for rejecting nearby interference in MEG measurements,” *Phys. Med. Biol.*, vol. 51, pp. 1–10, 2005.

[3] S. Taulu and R. Hari, “Removal of magnetoencephalographic artifacts with temporal Signal-Space Separation: Demonstration with single-trial auditory-evoked responses,” *Hum. Brain Mapp.*, vol. 1534, no. 30, pp. 1524–1534, 2009.

[4] N. Tzourio-Mazoyer *et al.*, “Automated anatomical labeling of activations in SPM using a macroscopic anatomical parcellation of the MNI MRI single-subject brain.,” *Neuroimage*, vol. 15, no. 1, pp. 273–89, 2002.

[5] A. Hillebrand *et al.*, “Direction of information flow in large-scale resting-state networks is frequency-dependent,” *Proc. Natl. Acad. Sci.*, vol. 113, no. 14, pp. 3867–3872, 2016.

[6] A. Hillebrand, K. D. Singh, I. E. Holliday, P. L. Furlong, and G. R. Barnes, “A new approach to neuroimaging with magnetoencephalography,” *Human Brain Mapping*, vol. 25, no. 2. pp. 199–211, Jun-2005.

[7] A. Hillebrand and G. R. Barnes, “Beamformer analysis of MEG data,” *International Review of Neurobiology*, vol. 68. pp. 149–171, 2005.

[8] T. Sekihara and S. Kumano, “Determination of compositeness of the Λ (1405) resonance from its radiative decay,” *Phys. Rev. C - Nucl. Phys.*, vol. 89, no. 2, pp. 1–12, 2014.
